# A comparative evaluation of the generalised predictive ability of eight machine learning algorithms across ten clinical metabolomics data sets for binary classification

**DOI:** 10.1007/s11306-019-1612-4

**Published:** 2019-11-15

**Authors:** Kevin M. Mendez, Stacey N. Reinke, David I. Broadhurst

**Affiliations:** Centre for Metabolomics & Computational Biology, School of Science, Edith Cowan University, Joondalup, 6027 Australia

**Keywords:** Metabolomics, Partial least squares, Support vector machines, Random forest, Artificial neural network, Machine learning, Jupyter, Open source

## Abstract

**Introduction:**

Metabolomics is increasingly being used in the clinical setting for disease diagnosis, prognosis and risk prediction. Machine learning algorithms are particularly important in the construction of multivariate metabolite prediction. Historically, partial least squares (PLS) regression has been the gold standard for binary classification. Nonlinear machine learning methods such as random forests (RF), kernel support vector machines (SVM) and artificial neural networks (ANN) may be more suited to modelling possible nonlinear metabolite covariance, and thus provide better predictive models.

**Objectives:**

We hypothesise that for binary classification using metabolomics data, non-linear machine learning methods will provide superior generalised predictive ability when compared to linear alternatives, in particular when compared with the current gold standard PLS discriminant analysis.

**Methods:**

We compared the general predictive performance of eight archetypal machine learning algorithms across ten publicly available clinical metabolomics data sets. The algorithms were implemented in the Python programming language. All code and results have been made publicly available as Jupyter notebooks.

**Results:**

There was only marginal improvement in predictive ability for SVM and ANN over PLS across all data sets. RF performance was comparatively poor. The use of out-of-bag bootstrap confidence intervals provided a measure of uncertainty of model prediction such that the quality of metabolomics data was observed to be a bigger influence on generalised performance than model choice.

**Conclusion:**

The size of the data set, and choice of performance metric, had a greater influence on generalised predictive performance than the choice of machine learning algorithm.

**Electronic supplementary material:**

The online version of this article (10.1007/s11306-019-1612-4) contains supplementary material, which is available to authorized users.

## Introduction

The multidisciplinary field of *data science* is concerned with extracting insights from data using a diverse set of computational methodologies, theories, and technologies (Blei and Smyth 2017). Within data science, there are two competing scientific philosophies: *classical statistics* and *machine learning* (Breiman 2001b). Classical statistics aims to formalise relationships between dependent and independent variables based on a clearly defined set of assumptions from which mathematical models are parametrised. The aim is to derive meaningful statistical inference (properties of an underlying probability distribution) for the measured variables, assuming that the observed data is sampled from a larger population. Conversely, machine learning uses ad-hoc computational algorithms that iteratively optimise (or ‘learn’) without necessarily relying on any formal statistical assumptions (Bishop 1995). Here, the aim is typically prediction rather than explanation, and inference is replaced by validation through testing the model with new data. Both approaches add insight into a given data set. Ideally, one would like a machine learning method that can be used for both prediction and statistical inference. Historically, for metabolomics (Gromski et al. 2015), that method has been partial least squares regression (PLS) (Wold 1975; Wold et al. 1993).

PLS has become the standard multivariate machine learning algorithm in metabolomics for several reasons. Firstly, PLS is a projection method, where highly multivariate data is projected into a smaller coordinate space (latent variables) before regressing to a dependent variable. This not only allows data sets with more variables than samples to be modelled without resorting to prefiltering variables (essential for hypothesis-generating experiments), it also plays to the strength of metabolomics, over other ‘omic platforms, in that there is inherently a large amount of inter-metabolite covariance in any biological system (Dunn et al. 2011), which is likely best represented as latent structure. Secondly, once optimised, a PLS model can be reduced to the form of a standard linear regression, from which inference about the importance of constituent metabolites can be made (Gromski et al. 2015). Finally, the algorithm is computationally inexpensive, and historically excellent software has been readily available through companies such as Umetrics (Umeå, Sweden) and Eigenvector Research (Washington, USA). This has accelerated its widespread adoption across the metabolomics community.

While easily interpretable PLS is inherently a linear algorithm, capable of modelling only linear latent covariance. As biological data are often non-linear (Mosconi et al. 2008) it is probable that metabolomics data also has a non-linear latent structure. As such, more complex non-linear machine learning methods such as random forest (RF), kernel support vector machine (SVM), and artificial neural networks (ANNs) may be more applicable for analysing metabolomics data. These alternative methods have spasmodically appeared in metabolomics literature, but never really gained much traction. This could be due to convoluted methods for determining metabolite inference, but equally because historically these methods have been computationally expensive, and software lacked widespread availability. As metabolomics experiments continue to become more complex in design, with increasingly large data sets, the opportunity to exploit concomitant advances in computational power and availability of open source software means that non-linear machine learning algorithms have become a viable alternative to PLS, particularly in situations where predictive performance is more important than inference.

The aim of this study was to compare the general predictive performance of an archetypal set of linear and non-linear machine learning algorithms evaluated across a representative number of clinical metabolomics data sets. The number of data sets was limited to ten and represented a cross section of current published data in terms of measurement instrument, number of samples, and complexity of biological question. This allowed the study to be small enough to be tractable (providing all data and code as interactive Jupyter notebooks) but also large enough to extract some general conclusions. We hypothesise that for binary classification using metabolomics data, non-linear machine learning methods will provide superior generalised predictive ability when compared to linear alternatives, in particular when compared with the current gold standard PLS discriminant analysis.

It is important to note that it is **not** the aim of this study to challenge the published results related to these data sets, or to pitch data sets against each other. All interpretations should be based only on the relative performance of competing algorithms for a given data set, and then a generalised meta-analysis of performance rankings across data sets. Also, the aim is to compare predictive performance, not metabolite inference, thus no biological interpretation of models is considered.

## Methods

### Data sets

The following criteria were used to identify ten metabolomics data sets for this comparative evaluation:

1. Data were of clinical origin.
2. Data were previously published.
3. Data publicly available at either *MetaboLights* or *Metabolomics Workbench* data repositories (www.ebi.ac.uk/metabolights; www.metabolomicsworkbench.org)
4. Metabolite data available in a form amenable for direct modelling (All feature selection/deconvolution performed and the resulting data matrix available in either a flat text file or common format of spreadsheet—e.g. Microsoft Excel).
5. Experimental data (e.g. Clinical Outcome) available in a form amenable for direct modelling.
6. A clear binary outcome available to model (either a primary or secondary outcome of the publication, or a subset of a multi-class study) and the number samples in each class are reasonably balanced.
7. Data representative of the three primary metabolomics technologies (nuclear magnetic resonance; gas chromatography mass spectrometry; liquid chromatography mass spectrometry).
8. Data representative of multiple biofluids (e.g. blood, urine, faeces).
9. A range of samples sizes (from less than 50 to more than 500).

The computational framework for this study (Sect. 2.4) required data to be converted to a standardised Microsoft Excel file format, using the Tidy Data framework (Wickham 2014), where each variable forms a column, each observation forms a row, and each type of observational unit forms a table. To this end, for each study, data are split into two linked tables. The first, named **Data**, contains data values related to each observation. i.e. metabolite concentrations $M_{1} \ldots M_{n}$, together with metadata such as: *injection order*, *sample type*, *sample identifier* and *outcome class*. The second table, named **Peak**, contains data that links each metabolite identifier ($M_{i}$) to a specific annotation (metabolite name) and optional metadata (e.g*. mass, retention time, MSI identification level, number of missing values, quality control statistics*). Standardising the data format before data analysis enabled clear presentation, and efficient reuse, of computer code.

### Machine learning algorithms

The following eight machine learning methods were considered for this study:

1. Partial least squares regression (a.k.a. projection to latent structures).
2. Principal components regression.
3. Principal components logistic regression.
4. Linear kernel support vector machines.
5. Radial basis function kernel support vector machines.
6. Random forests.
7. Linear artificial neural networks.
8. Non-linear artificial neural networks.

All methods were implemented in the Python programming language using standard packages where possible. Python packages: *Sci*-*kit learn* (Pedregosa et al. 2011), *Numpy* (Kristensen and Vinter 2010), *Pandas* (McKinney 2010), *Bokeh* (Bokeh-Development-Team 2018), *Keras* (Chollet 2015), *Theano* (Theano-Development-Team 2016). Details are provided in the supplementary files.

Before providing a brief overview of each method it is important to understand the concept of a *hyperparameter*. In machine learning, a hyperparameter is a parameter that is used to either configure the structure of the underlying model or the characteristics of the learning process. Its value is fixed before the learning process begins. All other parameters (coefficients, or weights) are determined through the training process. Different algorithms require different, and possibly multiple hyperparameters. Some simple algorithms (such as logistic regression) require none, many require only one (PLS requires only the optimisation of the number of latent variables), and others (such as artificial neural networks and random forests) require many. The number, type and function are described below.

#### Partial least squares regression

Partial least squares regression (PLS) (Wold 1975; Wold et al. 1993) is a widely used technique for constructing predictive models with metabolomics data (Gromski et al. 2015) (Broadhurst and Kell 2006), especially when the number of independent variables (metabolites) is much larger than the number of data points (samples). PLS uses the *projection to latent space* approach to modelling the linear covariance structure between two matrices (${\mathbf{X}}$ and ${\mathbf{Y}}$). A PLS model will try to find the multidimensional direction in the ${\mathbf{X}}$ space that explains the maximum multidimensional variance direction in the ${\mathbf{Y}}$ space. In lay terms: if the ${\mathbf{X}}$ matrix is thought of as a set of $N$ data points in $M$-dimensional space (where, $N$ is the number of samples and $M$ is the number of metabolites), and ${\mathbf{Y}}$ is a binary vector, length $N$, describing the classification of samples (e.g. case = 1 & control = 0), then PLS rotates and projects those data points into a lower dimensional space (typically 2 or 3 dimensions) such that discrimination (covariance) between the two labelled groups in the subspace is maximised.

Classification PLS is generally referred to as PLS *discriminant analysis* (PLS-DA). Importantly, PLS-DA is considered a linear regression method as the final predictive model can be reduced to the standard linear form $y^{*} = \beta_{0} + \beta_{1} x_{1} + \beta_{1} x_{2} + \ldots + \beta_{n} x_{N}$, where $\beta_{0} \ldots \beta_{N}$ is a vector of PLS coefficients and $y^{\varvec{*}}$ is the model prediction (typically, we define a positive classification if $y^{*} > 0.5$ and a negative classification if $y^{*} < 0.5$). For this study, each PLS model was optimised using the SIMPLS algorithm (de Jong 1993). PLS models have a single tuning hyperparameter: the number of latent variables (i.e. the number discriminant dimensions the ${\mathbf{X}}$ matrix is projected).

#### Principal component regression

Principal component regression (PCR) (Hastie et al. 2009; Jolliffe 1982) was a mathematical precursor to PLS. It builds upon the widely used multivariate descriptive statistical model: principal components analysis (PCA) (Jolliffe 2002). In PCA the ${\mathbf{X}}$ matrix is rotated and projected into a lower dimensional space based on orthogonal covariance, such that principal component 1 (PC_1_) describes the direction of maximal variance in ${\mathbf{X}}$, principal component 2 (PC_2_) describes the second orthogonal direction of maximal variance, PC_3_ the third direction … etc. PCA is converted into a predictive model by using the principal components as independent variables, and ${\mathbf{y}}$ as the dependent variable, in a multiple linear regression (MLR), with coefficients estimated by the least-squares method (Seber 2004). As with PLS, PCR is considered a linear regression method as the independently calculated PCA + MLR coefficients can be combined and reduced to the standard linear form $y^{*} = \beta_{0} + \beta_{1} x_{1} + \beta_{1} x_{2} + \ldots + \beta_{n} x_{N}$, where $\beta_{0} \ldots \beta_{N}$ is a vector of PCR coefficients and $y^{\varvec{*}}$ is the model prediction (typically, we define a positive classification if $y^{*} > 0.5$ and a negative classification if $y^{*} < 0.5$). PCR models have a single tuning hyperparameter: the number of principal components to use in the MLR.

#### Principal component logistic regression

PLS and PCR are usually solved by minimizing the least squares error of the model fit to the data. As such, errors are penalized quadratically. The underlying assumption of this method is that model residuals are normally distributed ($\varvec{y} - \varvec{Xb} = N\left({0,\sigma} \right)$). For a binary classification problem this may not be a valid (or useful) assumption. Consider a model for categorical outcomes ($y \in \left\{{0,1} \right\}$), where we define a positive classification if $y^{*} > 0.5$ and a negative classification if $y^{*} < 0.5$. If the model predicts the outcome to be 23 when truth is 1, or the model predicts the outcome to be − 43 when the truth is 0, nothing has been lost. Having an extremely large absolute error of prediction is not detrimental to the classification. However, least squares regression will consider this error important (remember all errors are penalized quadratically) and try to reduce it—unnecessarily. An alternative modelling technique is to make the binary outcome prediction a probability of correct classification, rather than a regression. To do this we use logistic regression. For logistic regression, observations $y \in \left\{{0,1} \right\}$ are assumed to follow a Bernoulli distribution, and uses a logistic loss function to model the dependent variable. The logistic function acts as a squashing function for extreme positive or negative values, causing large errors to be penalized asymptotically to a constant value (Menard 2002).

Accordingly, principal component logistic regression (PCLR) differs from PCR only in the change in loss function (logistic rather than quadratic), which can be visualised as a linear regression pushed through a logistic transformation (squashing function). So for PCLR, PCA is converted into a predictive model by using the principal components as independent variables, and $\varvec{y}$ as the dependent variable ($y \in \left\{{0,1} \right\}$), in a logistic regression (LR), with coefficients estimated using the maximum likelihood method (Menard 2002). PCLR is also considered a linear regression method as the independently calculated PCA + MLR coefficients can be combined and reduced to a model that is “linear in the coefficients” of the form $ln\left({\frac{{p_{+}}}{{1 - p_{+}}}} \right) = \beta_{0} + \beta_{1} x_{1} + \beta_{1} x_{2} + \ldots + \beta_{n} x_{N}$, where $\beta_{0} \ldots \beta_{n}$ is a vector of PCLR coefficients and $p_{+}$ is the predicted probability of positive outcome. PCLR models have a single tuning hyperparameter: the number of principal components to use in the MLR.

#### Linear kernel support vector machines

The objective of the linear kernel support vector machine (SVM-Lin) algorithm is to find a hyperplane in an *M*-dimensional space (*M *= the number of features) that distinctly classifies the *N* data points in the ${\mathbf{X}}$ matrix ($N \times M$). To separate two classes of data points, there are many possible hyperplanes that can be chosen. The role of the SVM algorithm is to determine the direction (or rotation) of the hyperplane that maximises the margin of discrimination (i.e. the distance between the closest data points at the edge of each class is made as large as possible). The support vectors are the data points that best define this margin. Importantly, and what makes SVM unique, is the process of maximising the margin makes the SVM robust to correctly classifying new data that may lie within that margin either side of the classification hyperplane (acting like a classification buffer). The loss function that enables SVM to maximize the margin is called the *hinge loss* function. SVM-Lin models have a single tuning hyperparameter called the regularization parameter (termed *C* for ‘cost’ by the Python library used in this study). The regularization parameter allows some flexibility regarding the number of misclassifications made by the hyperplane margin (and can be thought of as the degree in which the buffer of a given thickness is enforced—Supplementary Fig. 1). For a large value of C, the SVM will choose a small margin for the hyperplane if that hyperplane does a better job of getting all the training points classified correctly (hard margin). Conversely, a small value of C will cause the SVM to optimise to a larger margin separating hyperplane, even if that hyperplane misclassifies more points (soft margin). This regularisation is very important for allowing the SVM to generalise well and not over inflate the importance of individual data points in the optimisation process. An excellent detailed, and more mathematical, explanation of SVM is provided by Steinwart and Christmann (2008).

#### Radial basis function kernel support vector machines

SVMs can also be configured to perform non-linear classification by implicitly mapping input data into a high-dimensional feature space. This process is known as the *kernel trick*. The idea is to gain linearly separation by mapping the data to a higher dimensional space (see Supplementary Fig. 2). There are many kernel functions available, but the most popular is the *radial basis function* (RBF). An RBF, $\varphi \left({x,y} \right)$, maps the distance between two points into the range $\left[{0,1} \right]$ using a nonlinear transformation such that $\varphi \left({x,y} \right) = \varphi \left({\parallel x - y\parallel} \right)$. The standard RBF function is the Gaussian function: $\varphi \left({x,y} \right) = e^{{- \left({\gamma \parallel x - y\parallel} \right)^{2}}}$, where $\gamma$ is a shaping parameter to be tuned. The optimisation process for SVM-RBF is then identical to the SVM-Lin except now the optimal linear hyperplane is found with the assistance of the additional radial dimension, equivalent to a nonlinear hyperplane in the original data space. SVM-RBF models have two tuning hyperparameters: (i) the regularization parameter *C* (as described in 2.2.3) and (ii) the gaussian shape parameter, $\gamma$. If $\gamma$ is large the Gaussian shape is very tight leading to over-fitting. Conversely, if $\gamma$ is very small, the transformation is ineffective. The two hyperparameters are somewhat interdependent. A small value of C can compensate for a large value for $\gamma$. An excellent explanation of kernel methods applied to SVM is provided by Schölkopf and Smola (2001).

#### Random forests

Random Forest (RF) classifiers are radically different to the other ML methods used in this study. They are a type of ensemble classifier, where multiple *base classifiers* are trained and then aggregated to generate a single prediction. To avoid strong correlation between base classifiers, which in turn leads to overfitting, each base classifier must be unique, and thus differ in either the algorithm used, hyperparameter settings, or the training data. With RFs the base classifier is a *decision tree*. Thus, we are dealing with an ensemble of many decision trees (a forest of random decision trees).

A decision tree is top-down hierarchical structure of nodes connected by branches visualised as an inverted tree (Supplementary Fig. 3). Each node contains a logical question that sends a sample down one of two branches (a binary split), which in turn leads to another node, and on, and on, until it reaches a terminal node, which will provide a predicted classification. For example, to classify a new sample (say, based on a metabolite profile of 300 metabolites: $m_{1} \ldots m_{300}$) we start at the *root node* and performs the split described therein (e.g. **if** $m_{5} > 52$ **then** *Branch 1*, **else** *Branch 2*). Depending on the result we then descend the tree to the next *internal node* (e.g. **if** $m_{254} > 22$ **then** *Branch 3*, **else** *Branch 4*). Eventually we reach a *leaf node* at which time a classification is made (e.g. **if** $m_{42} > 12$ **then** *Case*, **else** *Control*). The result is a complex, but intuitive, multivariate binary-logic based predictive classification algorithm. However, inherently, the deeper the tree the fewer data points are used to split the samples into different classes, and as such they are prone to overfitting unless very large data sets are employed.

Random forest classifiers aggregate multiple trees (typically 100+ trees) to ameliorate the overfitting problem. Specifically, it uses Classification and Regression Tree (CART) optimisation (Breiman et al. 1984). The algorithm also reduces the previously mentioned correlation issue by allowing only a random subset of features on which to base the split at each node (typically the number in this subsample is equal to the square root of the total number of available features). To avoid any additional overtraining, trees can be constrained to a maximum depth and, during training, the minimum number of samples at each split and a minimum number of samples at each leaf node can be fixed. It has been shown that averaging the classification across many overtrained shallow CARTs produces a robust multivariate classifier (Breiman 2001a). For this comparative study using metabolomics data our preliminary analysis showed that varying many of the hyperparameters had minimal impact on final RF performance (i.e. ‘number of trees’; ‘number of features sampled during training’; ‘minimum number of samples at each split’), thus they were kept constant at their default values. This reduced the number of tuneable hyperparameters to: (i) *tree depth*, and (ii) *minimum number of samples classified at each leaf node during training (percentage)*.

#### Linear artificial neural network

Artificial neural networks (ANNs), inspired by the biological interconnections in the brain, consist of a layered weighted network of interconnected mathematical operators (neurons). The most common ANN is the feed-forward neural network. Here, each neuron acts as a weighted sum of the outputs of the previous layer applied multiplied to an activation function (typically linear or logistic function). Thus, a neuron with a linear activation is equivalent to a multiple linear regression, and a neuron with a logistic activation function is equivalent to logistic regression. A two-layer ANN (Supplementary Fig. 4) with a small number of linear neurons in the 1st layer (hidden layer) and a single linear neuron in the 2nd layer (output layer) is mathematically equivalent to PLS-DA, PCR. Moreover, a two-layer ANN with a small number of linear neurons in the hidden layer and a single logistic neuron in the output layer is mathematically equivalent to PCLR.

During ANN training, the interconnection weights between each layer of neurons (equivalent to coefficients in a regression) are iteratively optimised in a two-phase cycle. Firstly, data is projected through the model to generate a prediction (forward propagation), after which an error term is calculated based on the difference between the target and predicted outputs for all available data. This error is then projected back through the network, and individual weights are adjusted along the way (backward propagation). The aim is to optimise the classification performance by minimising misclassification using an appropriate loss function. For binary classification the best ANN loss function is cross-entropy: $loss = - \left({y \times ln\left({p_{+}} \right) + \left({1 - y} \right) \times ln\left({1 - p_{+}} \right)} \right)$ where $p_{+}$ is the predicted probability of positive classification and $y$ is the expected binary outcome. For ANN this loss function is then optimised using a gradient descent method (calculating the local loss function gradient and adjusting weights accordingly). The effectiveness of these methods is dependent on parameters that determine the rate and momentum of traversing the local error gradients (specifically ‘learning rate’, ‘momentum’, and ‘decay’ of the learning rate over time). This unique training method, known as backpropagation, allows for flexibility of ANN network architectures and a multitude of activation functions. For a detailed introduction to feedforward ANN please refer to Bishop (1995). When many layers on neurons are stacked in sequence the ANN is known as deep learning. Deep learning networks are beyond the scope of this study, but clearly warrant further investigation.

For this comparative study, a linear two-layer ANN with a small number of linear neurons in the hidden layer and a single logistic (sigmoidal) neuron in the output layer (ANN-LS) was implemented using stochastic gradient descent, with a binary cross-entropy loss function. Preliminary explorative analysis indicated that hyperparameters: *momentum*, and *decay*, could be set to a constant value (0.5 and 0 respectively) with little variation on performance. The hyperparameters *epochs* (number of training iterations), and *learning rate* are interdependent. Thus, we fixed the number of epochs (400) and varied the learning rate. This reduced the number of tuneable hyperparameters to: (i) the *number of neurons in the hidden layer*, and (ii) the *learning rate*.

#### Non-linear artificial neural network

To make the linear ANN into a non-linear ANN the hidden layer neurons can be changed to a non-linear activation function. In effect this is similar to the kernel trick described to SVM except the extra dimension is added to the latent variable space (hidden neuron space) rather than directly to the problem space. Although ANN with RBF hidden neurons were one of the first ever reported kernel methods (Broomhead and Lowe 1988; Park and Sandberg 1991) the more popular ANN with sigmoidal hidden neurons proved to be more effective (Bishop 1995; Wilkins et al. 1994). Thus, the final ML method in our collection is a two-layer ANN with a small number of sigmoidal hidden neurons and a single sigmoidal output neuron (ANN-SS) implemented using stochastic gradient descent, with a binary cross-entropy loss function. Again, the *momentum*, *decay* and *epochs* hyperparameters could be set to a constant value (0.5, 0, 400 respectively) without any detriment to performance. This reduced the number of tuneable hyperparameters to: (i) the *number of neurons in the hidden layer*, and (ii) the *learning rate*.

### Computational workflow

All workflows were implemented using the Python scripting language, presented in the form of interactive Jupyter notebooks following standard guidelines (Mendez et al. 2019a). All data and notebooks are publicly available on GitHub (https://cimcb.github.io/MetabComparisonBinaryML). Details of minor variations in the workflow for each individual model are provided at the top of each notebook (also provided in static html format as supplementary data). The standardised workflow for building, optimising, evaluating, and reporting each of the 80 models generated in this study is summarised below.

#### Splitting data into training and test sets

Multivariate predictive models are prone to overfitting. In order to provide some level of independent evaluation it is common practice to split the source data set into two parts: training data ($X_{train}$ and $Y_{train}$) and test data ($X_{test}$ and $Y_{test}$). The model is then optimised using the training data and independently evaluated using the test data. The true effectiveness of a model can only be assessed using the test data (Broadhurst and Kell 2006; Xia et al. 2013). It is imperative that both the training and test data are equally representative of the sample population, or else the test prediction will prone to sampling bias. For these workflows each data set is split with a ratio of 2:1 (2/3 training, 1/3 test) using stratified random selection. The data is split once and then applied to each ML method.

#### Optimisation

**Fig. 1:**
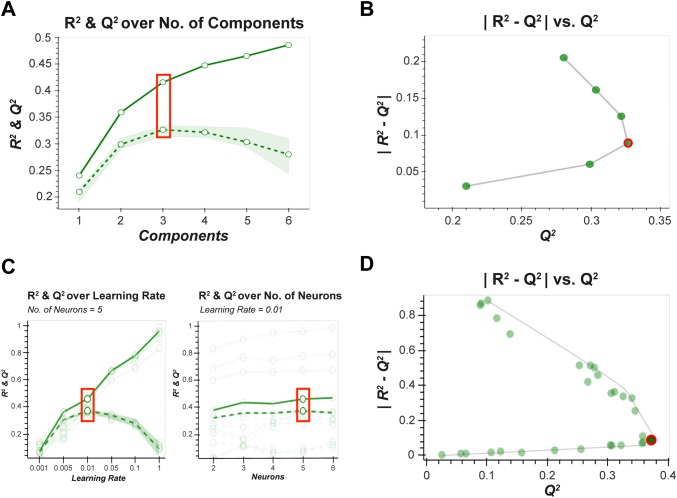
Hyperparameter optimisation. **a** An example of a standard $R^{2}$/$Q^{2}$ plot used for single hyperparameter optimisation (e.g. PLS). The optimum hyperparameter value (number of latent variables) indicated by the red square. **b** The corresponding generalised $\left| {R^{2} - Q^{2}} \right|\, vs. \,Q^{2}$ plot used for hyperparameter optimisation that is extended from (**a**), where the optimal number hyperparameter value (red circle) lies at the inflection of the data curve. **c** An example of a standard $R^{2}$/$Q^{2}$ plot used for multiple hyperparameter optimisation (e.g. ANN—one plot for “number of neurons” and another for “learning rate”). These plots are difficult to interpret as there are multiple curves for a given fixed value of the 1st hyperparameter across all the possible values of the 2nd hyperparameter. **d** The corresponding $\left| {R^{2} - Q^{2}} \right|\, vs. \,Q^{2}$ plot where each point corresponds to the evaluation for a pair of hyperparameter values. The optimal point, at the infection of the Pareto curve, is labelled as a red circle and this corresponds to the two red squares in (**c**), and optimal hyperparameter pair: number of neurons = 5 & learning rate = 0.01

Using the training data only, each model was optimised either using a linear search of a single hyperparameter, or a grid search of two hyperparameters, depending on the model type. Following fivefold cross-validation with 10 Monte Carlo repartitions (Broadhurst and Kell 2006; Hastie et al. 2009), plots of $\left| {R^{2} - Q^{2}} \right|\, vs. \,Q^{2}$ were generated to determine the optimal hyperparameter values (where *R*^*2*^ is the coefficient of determination for the full data set, and *Q*^*2*^ is the mean coefficient of determination for cross-validated prediction data across the 10 MC repartitions). The optimal hyperparameter was selected at the point of inflection of the outer convex hull of the $\left| {R^{2} - Q^{2}} \right|\, vs. \,Q^{2}$ data (i.e. Pareto optimization (Miettinen 1999)) (Fig. 1). If a clear inflection point was not present the hyperparameter (outcome) sitting on the Pareto front closest to the line $\left| {R^{2} - Q^{2}} \right| = 0.2$ was deemed optimal, based on the general rule that a difference between training and validation performance greater than 20% is indicative of overtraining (Eriksson et al. 2013). It has been previously shown (Szymańska et al. 2012) that for binary PLS-DA a more appropriate measure of performance is the area under the receiver operating characteristic curve (AUC). As such, plots of $\left| {AUC_{Full} - AUC_{CV}} \right|\, vs. \,AUC_{CV}$ were also provided and utilised as appropriate.

#### Model evaluation using test data

Using the optimal hyperparameters, a new model is fit using the training data only ($X_{train}$ and $Y_{train}$). When $X_{train}$ is applied to the model it produces a training prediction data ($Y_{train}^{*}$). The similarity of $Y_{train}$ to $Y_{train}^{*}$ gives an indication of training performance. The model is then independently evaluated by applying the test metabolite data ($X_{test}$; transformed and scaled using the metrics applied to $X_{train}$). This produces a test prediction ($Y_{test}^{*}$). The similarity of $Y_{test}$ to $Y_{test}^{*}$ gives an indication of test performance. For binary classification the best performance indicator is the receiver operator characteristic (ROC) curve (i.e. $ROC_{train}$, $ROC_{test}$) which can be further reduced to a single statistic using the area under the ROC curve (i.e. $AUC_{train}$, $AUC_{test}$).

#### Generalised predictive ability

Although the above ‘test data evaluation’ gives a good estimate of the true model performance when data sets are large, it potentially gives a biased estimate of performance when data sets are small. All sampled data sets are subject to sampling bias, such that they may not be truly representative of the generalised relationship being modelled (e.g. the metabotype for a specific disease). The smaller the sample data set the higher the probability of bias. This problem is only compounded when an already small sample is split into training and test data set. This bias can result in overly optimistic, or overly pessimistic evaluation, depending on the random chance of selecting an unrepresentative test set.

A measure of this uncertainty in prediction can be determined empirically by calculating confidence intervals of both the training and test evaluation metrics using bootstrap resampling (DiCiccio and Efron 1996; Efron 2000). The theoretical details of bootstrapping are beyond the scope of this paper. Briefly, this methodology allows accurate estimation of sampling distributions for almost any statistic by repeated random sampling. Each random sample selects ~ 2/3rd of the data points (called the in-bag sample, IB) leaving ~ 1/3rd (the out-of-bag sample, OOB). As such, bootstrapping can be useful for the evaluation of the optimal ML model configuration in metabolomics (Broadhurst and Kell 2006; Mendez et al. 2019b; Xia et al. 2013).

In this study, for each workflow, a model with the fixed optimal hyperparameter values (derived in 2.3.3) is retrained on data randomly sampled (IB sample) from the complete data set, and then evaluated on the unused data (OOB sample) for 100 resamples. This produces 100 different models, and therefore 100 IB predictions, and 100 OOB predictions. These predictions can then be translated into ROC curves from which 95% confidence interval can be calculated.

*Note*: The most effective way to get a true estimate of general performance is to ask a candidate model to predict scores for independently measured data (independent test data). Unfortunately, for the studies used in this paper, independent test data were unavailable. As such, the metrics presented are only **estimates**; however, the variability presented though confidence intervals allows some understanding of the uncertainty of any explicit single model performance metric, particularly when metrics are being compared across multiple competing ML algorithms (Xia et al. 2013).

## Results

### Data sets

The ten data sets curated for this study are described in Table 1. Six of the data sets were retrieved from *Metabolights* and four from *Metabolomics Workbench* data repositories. Six data sets acquired using LC–MS, two using NMR, and two using GC–MS. There was a cross section of biofluids (Plasma, Serum, Urine, Caecal, Saliva, Stool). The size of data set ranged from 59 to 968 subjects (data sets were reasonably balanced in outcome). Number of metabolites included in each data set ranged from 29 to 689. The outcome comparison (binary classification) performed is briefly described in the table and explained in detail at the top of each Jupyter notebook in the supplementary html files. Each data set was split into 2/3 training and 1/3 test using stratified random selection. The identical training and test sets were applied to each ML method so that comparison was unbiased.

**Table 1:** The ten data sets curated for this study

Study ID	Publication	Platform	Type	No. of samples (case/control)	No. of peaks	Case/control
MTBLS90^a^	Ganna et al. (2014); Ganna et al. (2015)	LC–MS	Plasma	968 (485/483)	189	Sex (M/F)
MTBLS92^a^	Hilvo et al. (2014)	LC–MS	Plasma	253 (142/111)	138	Breast cancer chemotherapy (before/after)
MTBLS136^a^	Stevens et al. (2018)	LC–MS	Serum	668 (337/331)	689	Postmenopausal hormone (estrogen/estrogen + progesterone)
MTBLS161^a^	Armstrong et al. (2015)	NMR	Serum	59 (34/25)	29	Chronic fatigue syndrome (case/control)
MTBLS404^a^	Thévenot et al. (2015)	LC–MS	Urine	184 (101/83)	120	Sex (M/F)
MTBLS547^a^	Zheng et al. (2017)	LC–MS	Caecal	97 (46/51)	42	High fat diet (case/control)
ST000369*	Fahrmann et al. (2015)	GC–MS	Serum	80 (49/31)	181	Adenocarcinoma (case/control)
ST000496*	Sakanaka et al. (2017)	GC–MS	Saliva	100 (50/50)	69	Debridement (pre/post)
ST001000*	Franzosa et al. (2019)	LC–MS	Stool	121 (68/53)	747	Inflammatory bowel diseases (Crohn’s disease/ulcerative colitis)
ST001047*	Chan et al. (2016)	NMR	Urine	83 (43/40)	149	Gastric cancer (gastric cancer/healthy)

*Indicates data sourced from Metabolomics Workbench (https://www.metabolomicsworkbench.org)

^a^Indicates data sourced from Metabolights (https://www.ebi.ac.uk/metabolights/)

### Comparative evaluation of generalised predictive ability across ML methods

**Fig. 2:**
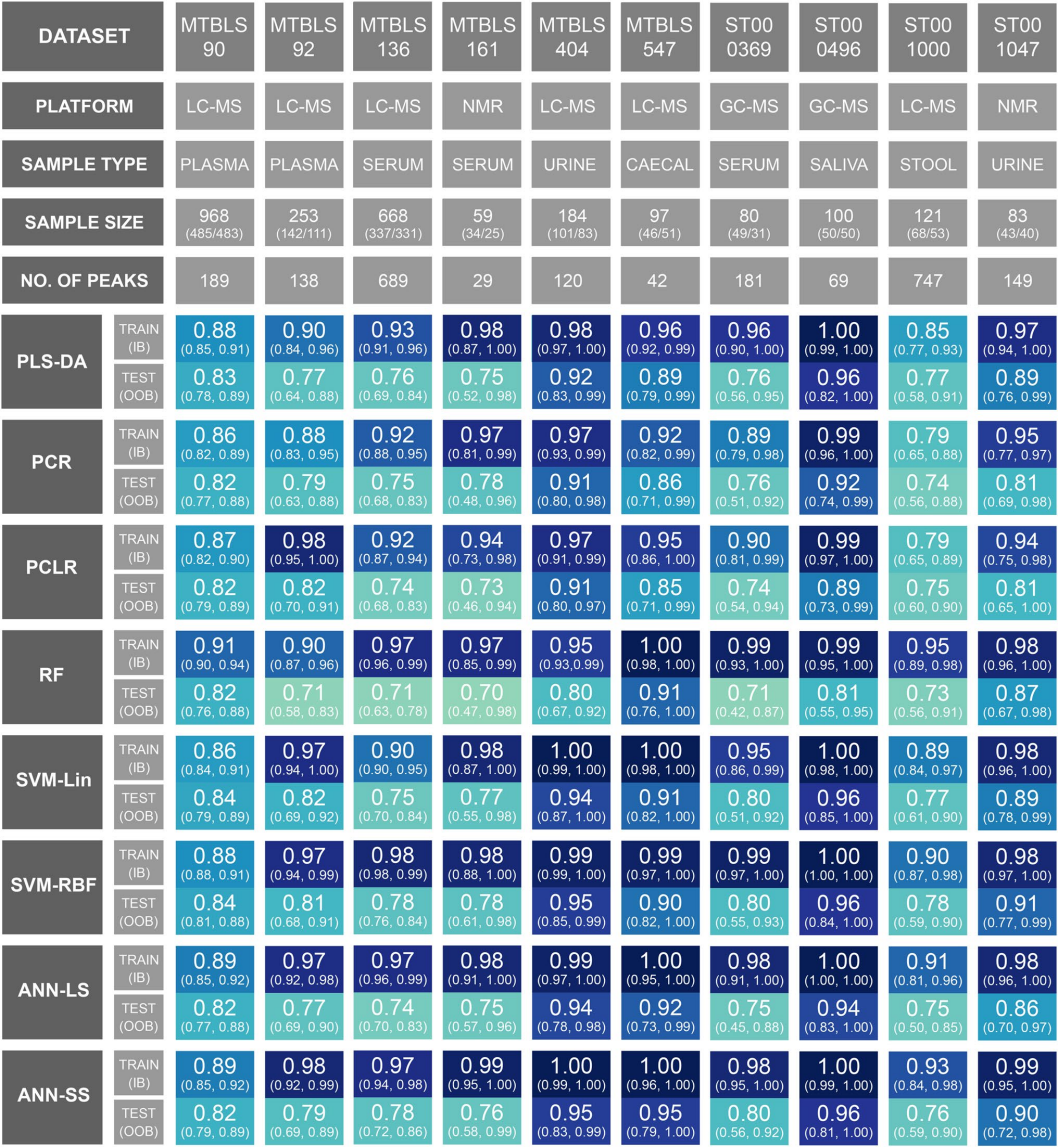
Bootstrap Model Performance. Training and test area under the Receiver Operator Characteristic curve (95% in-bag and out-of-bag bootstrap confidence intervals) for the complete matrix of datasets and machine learning methods

The hyperparameters for all 80 models were successfully optimised (see supplementary html files). For each optimally configured model, training/test data ROC curves was constructed and AUC_train_ / AUC_test_ calculated. Bootstrap resampling/retraining (n = 100) was performed and in-bag (IB) / out-of-bag (OOB) 95% confidence intervals were calculated. These results are presented as an annotated heatmap in Fig. 2. An interactive version of this figure linking each performance metric to a unique Jupyter notebook (including multiple statistics and visualisations) is available here: https://cimcb.github.io/MetabComparisonBinaryML/.

If the 95% confidence intervals are initially ignored, and the comparative evaluation across ML methods is based exclusively on the explicit test set predictions (AUC_test_), then SVM-RBF performs best across all data sets, closely followed by the nonlinear ANN-SS; however, the mean difference in AUC_test_ between SVM-RBF and ANN-SS across all data sets was only 0.004 (0.4%). The mean difference in AUC_test_ between SVM-RBF and PLS-DA was 0.02 (2%).

The mean difference in AUC_test_ between SVM-Lin and SVM-RBF was 0.006 (0.6%). The mean difference in AUC_test_ between ANN-LS and ANN-SS was 0.023 (2.3%).

**Fig. 3:**
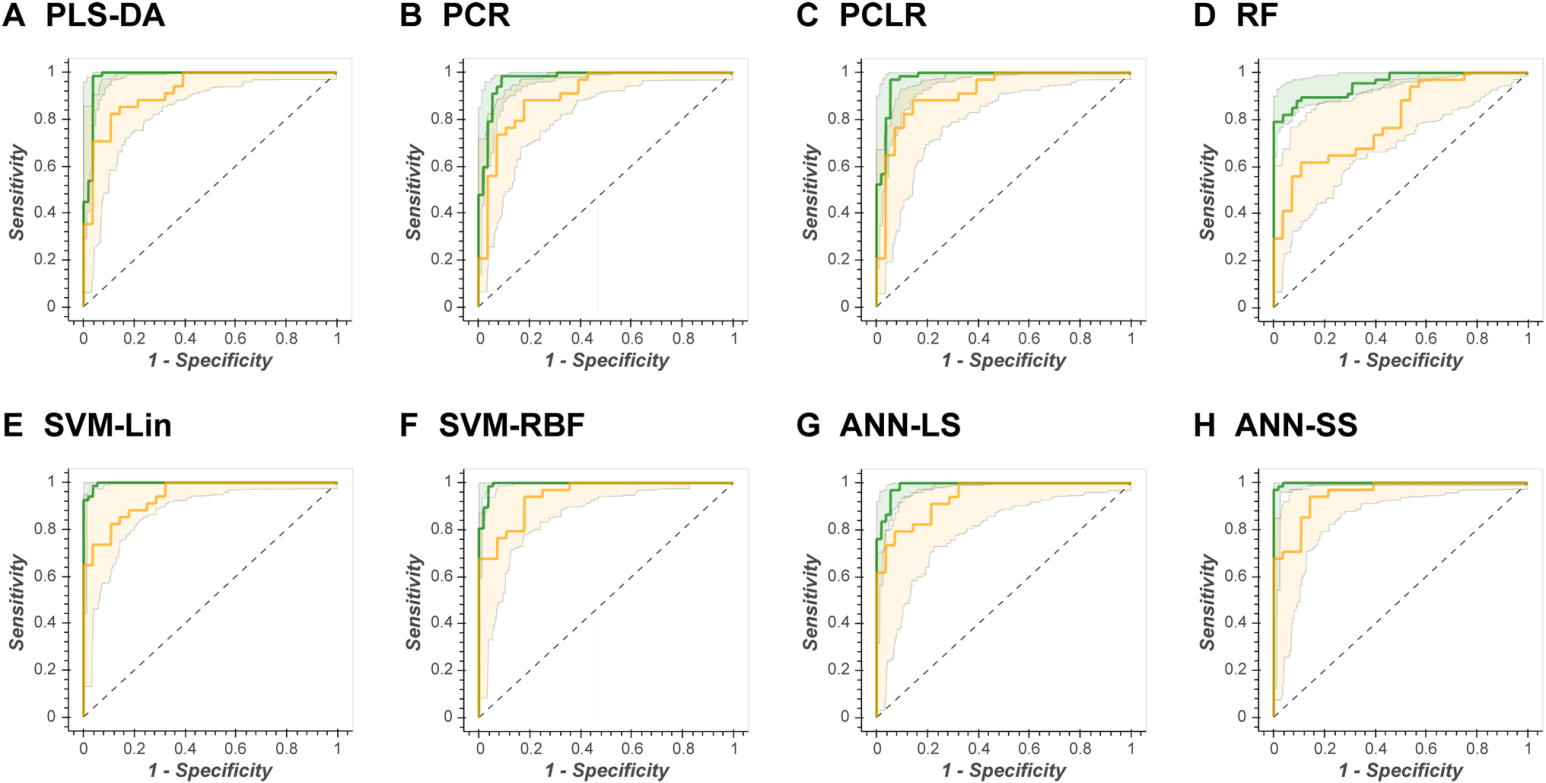
Illustration of the similarity of test prediction across all ML algorithms. The complete set Receiver Operator Characteristic curves for Data Set MTBLS404. Green line = ROC_train_, green shading = in-bag 95% confidence interval, yellow line = ROC_test_, yellow shading = out-of-bag 95% confidence interval. This resulted in: **a** AUC_test_ = 0.92; **b** AUC_test_ = 0.91; **c** AUC_test_ = 0.91; **d** AUC_test_ = 0.80; **e** AUC_test_ = 0.94; **f** AUC_test_ = 0.95; **g** AUC_test_ = 0.94; **h** AUC_test_ = 0.95

When the OOB 95% confidence intervals is used for test prediction then no single ML method is superior. ANN-LS, ANN-SS, SVM-Lin, SVM-RBF, and PLS-DA have very similar confidence intervals for each data set (for example, Fig. 3 shows the complete set of ROC curves for data set MTBLS404).

**Fig. 4:**
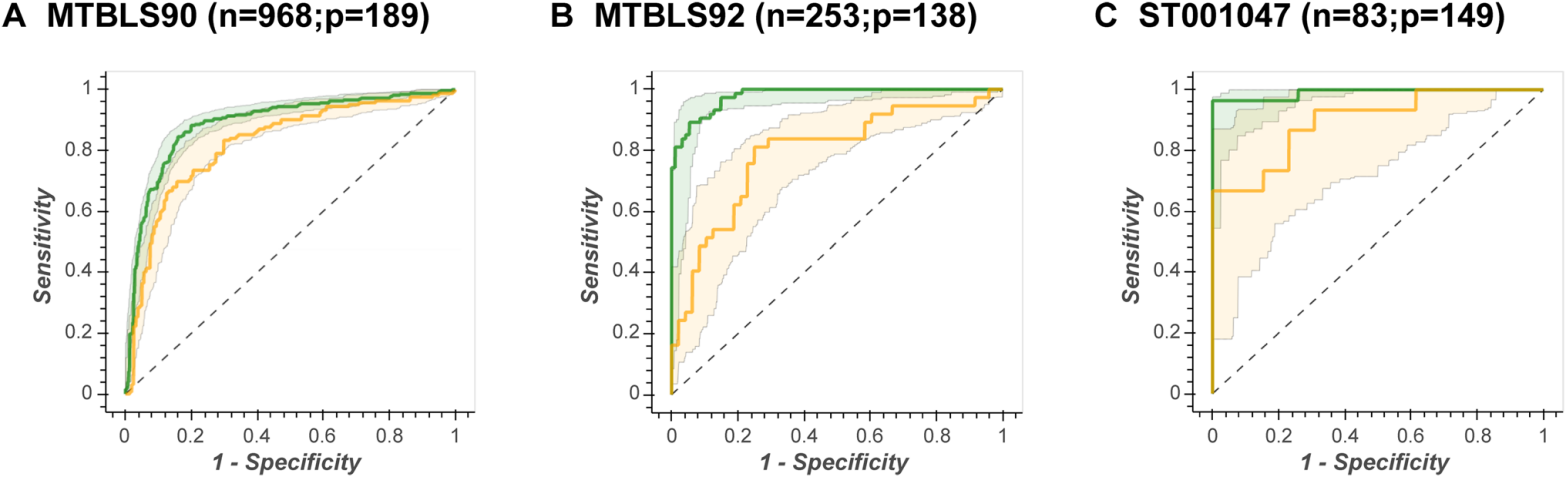
Inverse correlation between sample size and confidence intervals of models. SVM-RBF Receiver Operator Characteristic curves for three different size data sets (n = 968, n = 235, and n = 83). Green line = ROC_train_, green shading = in-bag 95% confidence interval, yellow line = ROC_test_, yellow shading = out-of-bag 95% confidence interval

If a single ML method is compared across multiple data sets, there is an observable inverse correlation between sample size and OOB 95% confidence interval (the fewer the samples the broader the confidence interval). This is illustrated in Fig. 4, where the ANN-SS ROC curves are presented for 3 different size data sets (n = 968, n = 235, and n = 83). Note that there is no observed correlation between performance and the number of metabolites modelled.

## Discussion

The primary hypothesis of this study was that for binary classification using metabolomics data, non-linear machine learning methods would provide superior generalised predictive ability when compared to linear alternatives, in particular when compared with the current gold standard partial least squares discriminant analysis (PLS-DA). Based on the ten data sets curated for this study, and the eight chosen machine learning methods, this primary hypothesis was disproved. Although support vector machines using a non-linear radial basis function kernel (SVM-RBF) and the fully sigmoidal feed-forward artificial neural network (ANN-SS) proved to be superior for all compared data sets with respect to AUC_test_, the difference in performance against their linear counterparts (ANN-LS and SVM-Lin) and PLS-DA was marginal once generalised confidence intervals were calculated. These results suggest that in general, for binary classification, metabolomics data is linearly separable, particularly when projected into a latent space. There is no need for the “kernel trick” described in Sect. 2.2.5. The poor overall performance of random forests (RFs) will be surprising to some, given claims that RFs cannot overfit. However, as Hastie et al. (2009) prove “when the number of variables is large, but the fraction of relevant variables small, random forests are likely to perform poorly with small m [number of samples]”. The inherent covariance in metabolomics data, which is an advantage to projection methods, hold no advantage for the random feature selection and data splitting performed by RF.

A second important observation from this study was that, despite standard k-fold cross-validation for optimisation, every model overtrained, and that the more complex the ML method the more severe the overtraining. This will be unsurprising to experts in the field, but it is worth noting. This is most strikingly observed in Fig. 2. The performance metrics (AUC_train_ & AUC_test_) for each model/data pair should, if not overtrained, be of the same value (same hue of blue in Fig. 3). Clearly, for several data sets the RF, SVM-RBF and ANN-SS are severely overtrained (reflected in differences between AUC_train_ and AUC_test_ of up to 25%). This is further illustrated in Fig. 3, where the in-bag ROC curves showed AUC_train_ > 0.98 for the PLS-DA, SVM-Lin, SVM-RBF and ANN-SS models applied to data set MTBLS404, but AUC_test_ were more conservative (0.92–0.95). As such, it is imperative that an estimate of generalised predictive ability is presented alongside any published model, preferably using an independently measured test data set or alternatively a methodology similar to the train/test or out-of-bag bootstrap method described herein. It is misleading to only present the confidence interval for the training data as a measure generalised prediction.

**Fig. 5:**
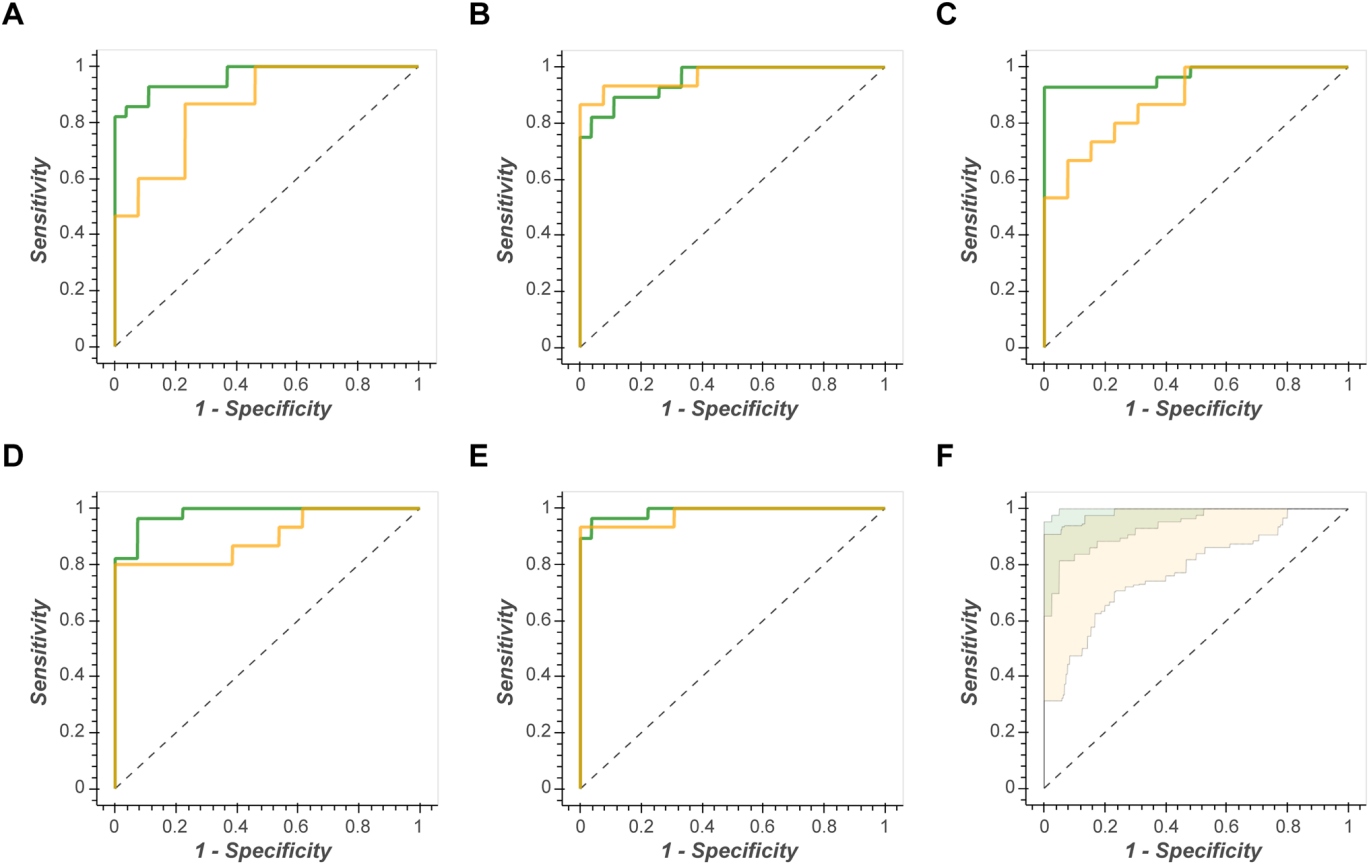
Prediction uncertainty when using train/test data splitting for validation. Receiver Operator Characteristic curves for training/test performance of PLS-DA on data set ST001047 for five iterations of stratified random splitting (2/3 training and 1/3 test). Green line = ROC_train_, yellow line = ROC_test_. This resulted in: **a** AUC_train_ = 0.96, AUC_test_ = 0.87; **b** AUC_train_ = 0.97, AUC_test_ = 0.96; **c** AUC_train_ = 0.97, AUC_test_ = 0.88; **d** AUC_train_ = 0.98, AUC_test_ = 0.90; **e** AUC_train_ = 0.99, AUC_test_ = 0.98. **d** The 95% OOB confidence interval for the same data. Note all **a**–**e** ROC_test_ curves lie within the 95% confidence interval

Thirdly, it is important to discuss the utility of calculating the bootstrap confidence interval for each candidate model configuration for the applied data. When data sets are small and potentially heterogeneous (as often observed in clinical studies) the use of random data splitting (e.g. 2/3 training, 1/3 test) to provide an unbiased performance evaluation can be dangerous. For truly unbiased evaluation the test set must exactly represent the training data. This may not be possible by random methods (even when stratified by outcome). This is illustrated in Fig. 5 where, for data set ST001047, the random split is repeated 5 times with dramatically different performance for a PLS-DA model using two latent variables. The bootstrap resampling enables the modeller to estimate this uncertainty. It is worth noting that for all 80 of the models presented in this paper the ROC_test_ curve lay within the bounds of the respective OOB 95% confidence interval (see supplementary notebooks). Even so, such bootstrapping provides only an estimate and care must be taken as there is a certain amount of data leakage as the same data that is being used to select the hyperparameters is being used to evaluate the model.

A final, but equally important, observation from this study was that the stability of a model was dependent on the number of samples available for training. This is best illustrated in Fig. 4. Here the generalised predictive ability of an ANN-SS model is compared across three data sets of increasing size. For data set ST001047 (n = 83) the out-of-bag ROC curves vary dramatically from AUC_OOB_ = 0.75–0.98). This implies that the underlying model parameters varying massively due to heterogeneity of the in-bag training sets. Which leads to the question: Is the complete data set a representative sample of the biological question? (in this case classifying gastric cancer). This phenomena, known as the *Rashomon Effect*, has been discussed at length by Breiman (2001b), Broadhurst and Kell (2006) and Broadhurst (2017). In contrast, data set MTBLS90 (n = 968) has extremely stable out-of-bag ROC curves implying that there is sufficient data to robustly model the biological question.

## Limitations of the study

While the results of this study will hopefully prove useful to the metabolomics research community, it is important to list some limitations. Firstly, focusing on binary classification we may have oversimplified the problem space. Non-linear ML methods may be more effective in multi-class problems, so results need to be interpreted with this in mind. Secondly, by focussing on published data there is a possibility that the results are biased (*publication bias*). All the data set used in this study were successfully published using a linear model. Given that, generally, only positive results are published it may be that, despite our best efforts, we did not have access to data sufficiently complex to require a non-linear model. Finally, the ML algorithms with more than two hyperparameters (i.e. ANN and RF) are presented in the Jupyter notebooks such that we limit the search strategy to a grid search of the two most sensitive hyperparameters, fixing the other hyperparameters at a constant value. A full parameter search was performed for each individual model under cross-validation conditions, and repeatedly the same hyperparameters had little effect on optimisation, so for clarity of presentation they were fixed at the same value across all data sets in the Jupyter notebooks provided. Interested readers are encouraged to download the data and notebooks and verify our findings.

## Conclusions

In this study of binary classification across ten publicly available metabolomics datasets we have shown that using non-linear machine learning showed no general improvement in predictability over linear methods. If we use the principle of Occam’s razor, where the simplest model wins out, PLS-DA remains a sensible first choice. However, improved computational power and open availability of high-quality software libraries means that comparing multiple models of a given data set is tractable. Our results clearly demonstrate that of equal importance to the choice of machine learning method is the way that each method is optimised, and how its generalised performance is evaluated. It is far too easy to overtrain a complex model and erroneously report misleading results. We have provided a generalised framework to investigate eight machine learning algorithms and a generalised optimisation and evaluation workflow that can be applied to any multivariate data with a binary outcome variable.

The likely most important conclusion from this study is a reiteration of the well-established machine learning trope *a model is only as good as the data that is used to train it*. We consider the 10 datasets used in this study are representative, both in sample size and scope, of biomarker studies published in metabolomics. The results presented here suggest that for robust predictive models the most important consideration is statistical power. There is no magic formula for calculating the number of samples needed for robust metabolomics multivariate machine learning, where estimates are dependent on many factors, including: the dimensionality of the data, the strength of effect, the degree of covariance (strength of latent structure), the heterogeneity of the sample population, the repeatability of the measurement instrument, and the complexity of the model. However, as pointed out by Breiman (2001b), the curse of dimensionality dictates that the expected generalization error is proportional to the complexity of the model and inversely proportional to the number of samples used to build the model. Thus, for high dimensional data a complex model trained on a small data set will tend to have poor generalised performance as a classifier. Put simply, the larger and better curated (cleaned and identified) the data set, the more amenable it will be to non-linear machine learning algorithms.

## Future perspectives

In order for machine learning to have a meaningful impact on metabolomics then larger data sets need to be collated, and those data have to be pass stringent quality control checks (Broadhurst et al. 2018). It is important to note that an increasing number of metabolomics researchers, particularly in the clinical domain, outsource metabolomics data acquisition. Companies such as Metabolon (https://www.metabolon.com/), Nightingale Health (https://nightingalehealth.com/), and Biocrates (https://www.biocrates.com/) have built business models that depend on providing high-quality fully annotated data sets in a format amenable for data science. Most large academic laboratories also provide some level of similar service. This is illustrated by the recent successful ring trial for the Biocrates AbsoluteIDQ p400HR assay (Thompson et al. 2019) which will allow data sets from multiple labs to be potentially combined into one data analysis. Other approaches to data fusion have most recently been reported in the American Journal of Epidemiology by Yu et al. (2019) “Consortium of Metabolomics Studies (COMETS) Metabolomics in 47 Prospective Cohort Studies”.

As machine learning methods get more complex the demands for data get greater. The recent successes of deep learning in image processing, peak deconvolution and metabolite identification (Mendez et al. 2019a) means it is likely that such methods will also be applied to predictive modelling. As a community it is important that mechanisms are put in place to avoid over optimistic reporting of results, and that it is not simply assumed that a complex model is the best model. There is an urgent need for transparent and consistent reporting of all aspects of the metabolomics study lifecycle. The metabolomics community has made substantial efforts to align with FAIR (Findable, Accessible, Interoperable, and Reusable) data principles by utilizing open data formats [e.g. mzXML (Pedrioli et al. 2004)], developing data repositories [e.g. MetaboLights (González-Beltrán et al. 2012) and Metabolomics Workbench (Sud et al. 2016)], and with online spectral reference [e.g. METLIN (Smith et al. 2005), mzCloud (https://www.mzcloud.org/), MassBank (Horai et al. 2010), GNPS (Wang et al. 2016)], and online databases for metabolite identification and biochemical association [e.g. HMDB (Wishart et al. 2018)]. However, significant efforts are required to find ways to make metabolomics data modelling FAIR. One such approach is through Jupyter notebooks (Mendez et al. 2019b). Hopefully, the 80 Jupyter notebooks provided for this study will help inspire more open reporting of predictive modelling in metabolomics (https://cimcb.github.io/MetabComparisonBinaryML).

## Electronic supplementary material

Below is the link to the electronic supplementary material.

Supplementary material 1 (DOCX 437 kb)

## Data Availability

The metabolomics and metadata used in this paper were retrieved from Metabolights (https://www.ebi.ac.uk/metabolights/) Project IDs: MTBLS90 MTBLS92 MTBLS136 MTBLS161 MTBLS404 MTBLS547 and Metabolomics Workbench (https://www.metabolomicsworkbench.org/) Project IDs: ST000369 ST000496 ST001000 ST001047. This data were converted from the original data format to a clean format compliant with the Tidy Data framework, this is available at the CIMCB GitHub project page: https://github.com/CIMCB/MetabComparisonBinaryML.

## Software availability

All software developed for this paper is available at the CIMCB GitHub project page: https://github.com/CIMCB/MetabComparisonBinaryML.
