# Supplementary material for: A comparative evaluation of the generalised predictive ability of eight machine learning algorithms across ten clinical metabolomics data sets for binary classification
Source: Metabolomics. 2019 Nov 15;15(12):150. doi: 10.1007/s11306-019-1612-4 (PMC6856029; doi:10.1007/s11306-019-1612-4)
Supplement: Supplementary file 1 — Supplementary material 1 (DOCX 437 kb) [file 11306_2019_1612_MOESM1_ESM.docx]

A Comparative Evaluation of the Generalised Predictive Ability of Eight Machine Learning Algorithms across Ten Clinical Metabolomics Data Sets for Binary Classification.

**Authors**

Kevin M Mendez^1^, Stacey N Reinke^1^, David I Broadhurst^1*^

^1^Centre for Metabolomics & Computational Biology, School of Science, Edith Cowan University, Joondalup, 6027 Australia

*Corresponding author:

email: d.broadhurst@ecu.edu.au

phone: +61 (0)8-6304-2705

**ORCIDs:**

Kevin M Mendez: 0000-0002-8832-2607

David I Broadhurst: 0000-0003-0775-9581

Stacey N Reinke: 0000-0002-0758-0330

List of supplementary Jupyter Notebook html files [Model]_[DataSet].html

ANNLinSig_MTBLS136.html; ANNLinSig_MTBLS161.html; ANNLinSig_MTBLS404.html; ANNLinSig_MTBLS547.html; ANNLinSig_MTBLS90.html; ANNLinSig_MTBLS92.html; ANNLinSig_ST000369.html; ANNLinSig_ST000496.html; ANNLinSig_ST001000.html; ANNLinSig_ST001047.html; ANNSigSig_MTBLS136.html; ANNSigSig_MTBLS161.html; ANNSigSig_MTBLS404.html; ANNSigSig_MTBLS547.html; ANNSigSig_MTBLS90.html; ANNSigSig_MTBLS92.html; ANNSigSig_ST000369.html; ANNSigSig_ST000496.html; ANNSigSig_ST001000.html; ANNSigSig_ST001047.html; PCLR_MTBLS136.html; PCLR_MTBLS161.html; PCLR_MTBLS404.html; PCLR_MTBLS547.html; PCLR_MTBLS90.html; PCLR_MTBLS92.html; PCLR_ST000369.html; PCLR_ST000496.html; PCLR_ST001000.html; PCLR_ST001047.html; PCR_MTBLS136.html; PCR_MTBLS161.html; PCR_MTBLS404.html; PCR_MTBLS547.html; PCR_MTBLS90.html; PCR_MTBLS92.html; PCR_ST000369.html; PCR_ST000496.html; PCR_ST001000.html; PCR_ST001047.html; PLSDA_MTBLS136.html; PLSDA_MTBLS161.html; PLSDA_MTBLS404.html; PLSDA_MTBLS547.html; PLSDA_MTBLS90.html; PLSDA_MTBLS92.html; PLSDA_ST000369.html; PLSDA_ST000496.html; PLSDA_ST001000.html; PLSDA_ST001047.html; RF_MTBLS136.html; RF_MTBLS161.html; RF_MTBLS404.html; RF_MTBLS547.html; RF_MTBLS90.html; RF_MTBLS92.html; RF_ST000369.html; RF_ST000496.html; RF_ST001000.html; RF_ST001047.html; SVMLin_MTBLS136.html; SVMLin_MTBLS161.html; SVMLin_MTBLS404.html; SVMLin_MTBLS547.html; SVMLin_MTBLS90.html; SVMLin_MTBLS92.html; SVMLin_ST000369.html; SVMLin_ST000496.html; SVMLin_ST001000.html; SVMLin_ST001047.html; SVMRBF_MTBLS136.html; SVMRBF_MTBLS161.html; SVMRBF_MTBLS404.html; SVMRBF_MTBLS547.html; SVMRBF_MTBLS90.html; SVMRBF_MTBLS92.html; SVMRBF_ST000369.html; SVMRBF_ST000496.html; SVMRBF_ST001000.html; SVMRBF_ST001047.html

Supplementary figures: pages 2-3


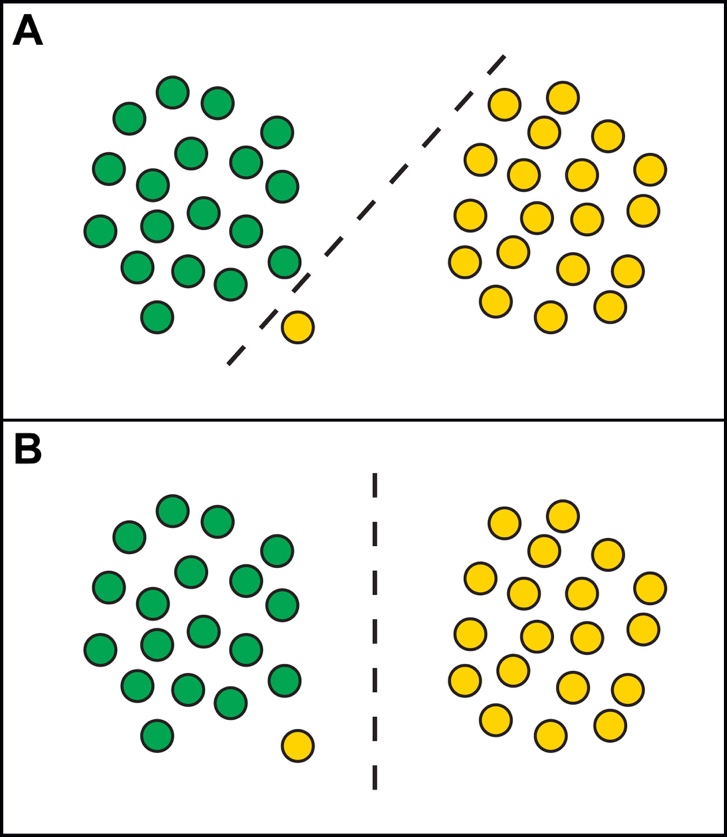


**Supplementary Figure 1:** Illustration of how the regularization parameter, C, in support vector machine (SVM) optimisation allows some flexibility regarding the number of misclassifications made by the hyperplane margin. For a large value of C (**A**), the SVM will choose a small margin for the hyperplane if that hyperplane does a better job of getting all the training points classified correctly (hard margin). Conversely, a small value of C (**B**) will cause the SVM to optimise to a larger margin separating hyperplane, even if that hyperplane misclassifies more points (soft margin).


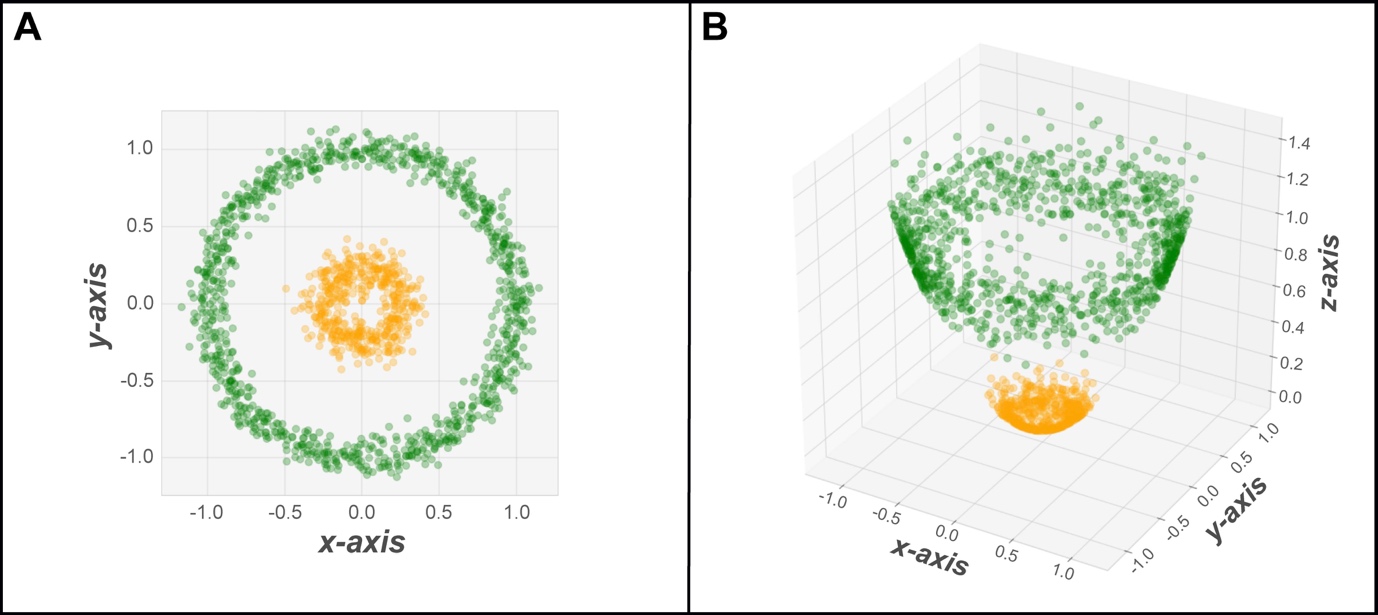


**Supplementary Figure 2:** SVMs can be configured to perform non-linear classification by implicitly mapping input data into a high-dimensional feature space. This process is known as the *kernel trick*. The idea is to gain linearly separation by mapping the original data (**A**) to a higher dimensional space (**B**). The data is now linearly separable in the z-axis.


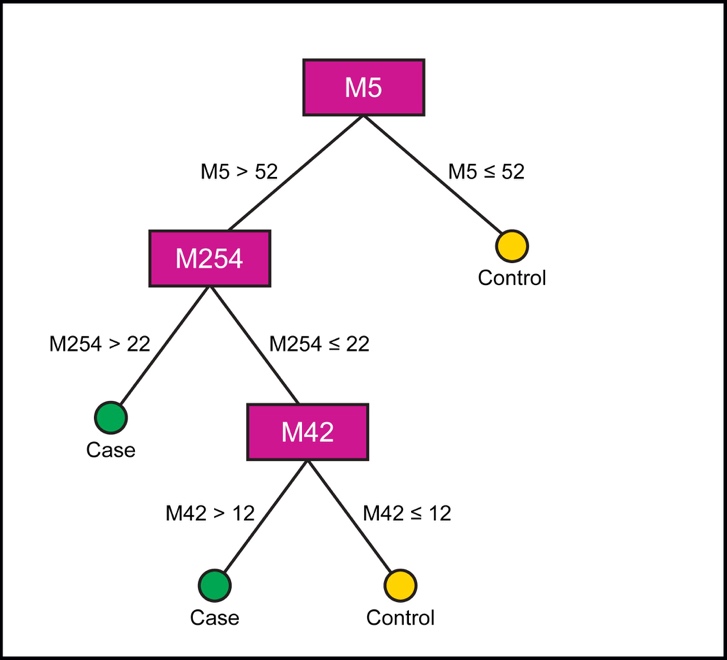


**Supplementary Figure 3:** A decision tree is top-down hierarchical structure of nodes connected by branches visualised as an inverted tree. Each node contains a logical question that sends a sample down one of two branches (a binary split), which in turn leads to another node, and on, and on, until it reaches a terminal node, which will provide a predicted classification. For example, to classify a new sample (say, based on a metabolite profile of 300 metabolites: $m_{1}\ldots m_{300}$) we start at the *root node* and performs the split described therein (e.g. **if** $m_{5}>52$ **then** *Branch 1*, **else** *Branch 2*). Depending on the result we then descend the tree to the next *internal node* (e.g. **if** $m_{254}>22$ **then** *Branch 3*, **else** *Branch 4*). Eventually we reach a *leaf node* at which time a classification is made (e.g. **if** $m_{42}>12$ **then** *Case*, **else** *Control*).

**
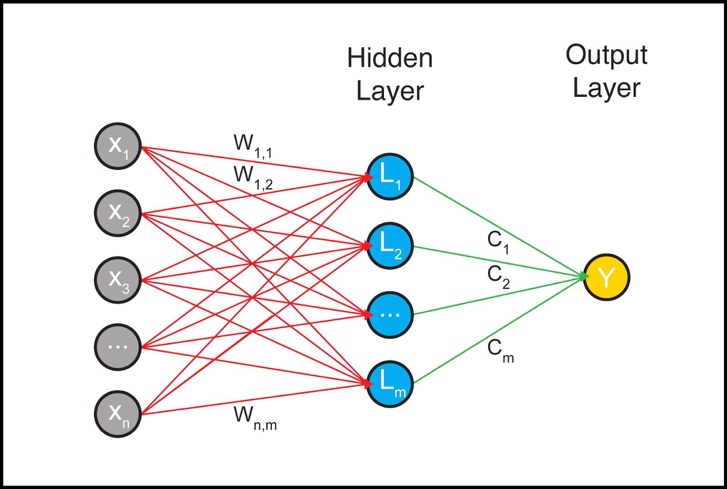
**

**Supplementary Figure 4:** A two-layer ANN (Supplementary Figure 4) with a small number of linear neurons in the 1^st^ layer (hidden layer) and a single linear neuron in the 2^nd^ layer (output layer)
